# Comparison of Two Different Pulsed Field Ablation Systems: The Dual Pulse System Study

**DOI:** 10.1111/jce.70078

**Published:** 2025-09-19

**Authors:** Behnam Subin, Corinne Isenegger, David Spreen, Philipp Krisai, Sven Knecht, Gian Völlmin, Nicolas Schaerli, Felix Mahfoud, Michael Kühne, Christian Sticherling, Patrick Badertscher

**Affiliations:** ^1^ Department of Cardiology University Hospital Basel Basel Switzerland; ^2^ Cardiovascular Research Institute Basel University Hospital Basel Basel Switzerland

**Keywords:** atrial fibrillation, catheter ablation, pulmonary vein isolation, pulsed field ablation

## Abstract

**Background:**

Recently, multiple pulsed field ablation (PFA)‐system were introduced for catheter ablation (CA) of atrial fibrillation (AF). However, data comparing procedural performance, the extent of low‐voltage areas (LVA), and myocardial injury between different PFA‐systems in a real‐world setting remain scarce.

**Methods:**

Consecutive patients undergoing CA for AF were enrolled. PFA was performed using either a Pentaspline catheter‐system (PCS) or a loop catheter‐system (LCS). The extent of acute antral LVA was assessed using a 3D‐electroanatomical mapping system. High‐sensitivity cardiac troponin T (hs‐cTnT) was measured the day after the procedure to assess myocardial injury.

**Results:**

A total of 120 patients (median age 67 [59–73] years, 29% female) underwent de novo pulmonary vein isolation (PVI). The PCS‐group included 90 patients, while the LCS‐group included 30 patients. Acute PVI was achieved in all patients (100%). Procedural times were significantly shorter in the PCS compared to the LCS‐group, including total procedure duration (57 [48–67] vs 66 [52–83] min, *p* = 0.016), left atrial dwell time (38 [32–48] vs 54 [38–65] min, *p* < 0.001), and ablation duration (17 [12–23] vs 24 [20–33] min, *p* < 0.001). Acute antral LVA and myocardial injury were significantly lower in the PCS compared to the LCS‐group (6.6 [5.0–8.9] vs. 19.2 [16.8–25.4] cm², *p* < 0.001 and hs‐cTnT of 1282 [892–1894] vs 1588 [1281–2110] ng/L, *p* = 0.029.

**Conclusion:**

Significant differences were observed between two commercially available PFA‐systems. While PCS was associated with significantly shorter procedural time, LCS resulted in a greater extent of acute antral LVA and myocardial injury levels.

## Graphical abstract

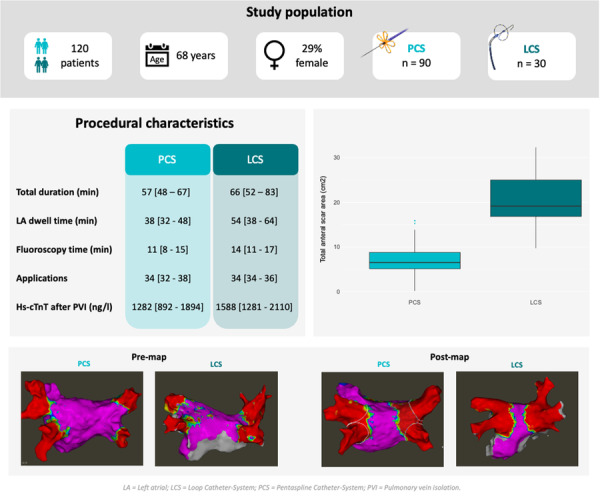

## 1 Introduction

Pulmonary vein isolation (PVI) represents the cornerstone of catheter ablation (CA) therapy for patients with atrial fibrillation (AF) [1]. Pulsed field ablation (PFA) is a novel, non‐thermal ablation technique in which high‐frequency electrical impulses induce myocardial tissue cell death. This ablation method is characterized by its high tissue specificity [2, 3, 4].

Recently, two PFA systems received FDA approval and are now commercially available: a pentaspline catheter‐system (PCS) and a loop catheter‐system (LCS). While multiple reports exist for each PFA system individually, comparative data between different PFA systems is scarce [5, 6, 7].

Both PFA systems are considered single‐shot devices and offer some key differences in technical features. First, the PCS is a 12 F over‐the‐wire multipolar ablation catheter with either a 31 or 35 mm diameter (FARAWAVE; Boston Scientific), consisting of 5 splines, each containing 4 electrodes (totaling 20 ablation electrodes). In contrast, the LCS is a 9 F over‐the‐wire multipolar ablation catheter (PulseSelect; Medtronic) with a 25 mm diameter loop integrated with 9 electrodes. Second, while the PCS can assume various shapes, including a spherical “basket” or a fully deployed flat “flower” configuration, the LCS differs in its design by allowing interchangeable configurations between a forward‐tilted lasso and a spiral shape. Third, the PCS delivers 5 packets of pulses over 2.5 s, which are not gated to the QRS complex, using field strengths of 1800, 1900, or 2000 volts. In contrast, the LCS, delivers four packets of pulses, each lasting 100–200 ms at 1500 V, gated to the QRS complex. Additional technical specifications for the PCS and LCS are listed in Table S1.

Whether these technical differences impact procedural characteristics, antral low‐voltage areas (LVA), or myocardial injury remains unclear. It has been well recognized that clinical outcomes after PVI may be linked to a wide antral approach compared to an ostial PVI [8]. Thus, a comparative study between different PFA systems is warranted.

This study aimed to compare two PFA systems regarding procedural characteristics, acute antralLVA, and myocardial injury in patients with AF undergoing de novo PVI.

## 2 Methods

### 2.1 Patient Characteristics and Study Design

This study included consecutive patients with paroxysmal or persistent AF from the prospective SWISS‐AF‐PVI registry (NCT03718364) who underwent a first‐time catheter ablation at the University Hospital Basel. After identifying the first 30 patients treated with the LCS, a group of 90 patients undergoing first‐time CA with the PCS during the same time frame was selected in a 1:3 ratio to establish two distinct groups for comparative analysis.

Exclusion criteria included a history of prior left atrial (LA) ablation and application of additional lesions such as posterior wall isolation. All patients were > 18 years old, provided written informed consent, and all patient information was anonymized. The study was conducted according to the principles of the Declaration of Helsinki and was approved by the local ethics committees.

### 2.2 Preprocedural Management

All patients underwent preprocedural transesophageal echocardiography to exclude the presence of LA thrombus. Additionally, each patient underwent supplemental imaging of the left atrium using either computed tomography (CT) or magnetic resonance imaging (MRI).

### 2.3 Pulmonary Vein Isolation

The procedural workflow of our institution has been previously described in detail [9, 10, 11].

In brief, all procedures were performed under deep sedation using midazolam, fentanyl, and continuous propofol infusion. Ultrasound‐guided double puncture of the right femoral vein was performed. A diagnostic catheter was introduced and positioned inside the coronary sinus, followed by a single transseptal puncture under fluoroscopic guidance. Heparin was administered to maintain an activated clotting time of ≥ 350 s. Intracardiac and surface electrograms were recorded at a speed of 100 mm/s (Sensis, Siemens, Erlangen, Germany).

PVI was performed using either a PCS or LCS as described below. Technical characteristics of both PFA systems are summarized in Supporting Information Table S1.

#### 2.3.1 Pentaspline Catheter System

For catheter ablation with the PCS (FARAWAVE), the target pulmonary vein was cannulated using a J‐tip guidewire (Amplatz ExtraStiff). The Pentaspline catheter, either 31 or 35 mm in size, was then advanced into the left atrium and positioned at the target vein through the 13‐French steerable sheath (FaraDrive). The device can be configured into two distinct poses: the flower and basket configuration. PFA was performed with four applications in each configuration per vein. A rotation of 30° to 40° was applied between each pair of PFA applications, after the two initial applications in each configuration. Additionally, two extra applications in the flower configuration were delivered at the carina of the right pulmonary veins. The ablation procedure was conducted at a voltage of 2 kV.

#### 2.3.2 Loop Catheter System

For catheter ablation with the LCS (PulseSelect), the target pulmonary vein was cannulated using a J‐tip guidewire (PV‐Tracker, Medtronic). Subsequently, the loop‐shaped ablation catheter was advanced over the wire at the ostium of the target vein. All applications were delivered using the circular configuration of the catheter; the spiral configuration was not used in this study. Each application consisted of four bipolar pulse trains delivered at a voltage of 1500 V. A total of four applications were administered in the ostial position and an additional four in the antral position. After each application, the catheter was rotated 90 degrees to achieve full circumferential isolation. The ablation procedure was conducted at a voltage of 1.5 kV.

#### 2.3.3 Mapping

Pre‐ and Postablation, a voltage map utilizing 3D‐EAM system (CARTO 3, Biosense Webster, Irvine, CA, USA) combined with a multipolar mapping catheter (Pentaray or Octaray, Biosense Webster) was created to guide PVI, assess first pass isolation rate, defined as acute isolation after performing standard applications for all PVs and to assess the acute ablation outcome by either PFA modality. Ablation was guided using the 3D mapping system, with PFA catheters being visualizedduring the procedure. All voltage maps were performed in sinus rhythm. For the left atrial voltage map, the bipolar voltage reference interval was set to 0.1–0.5 mV. The extent of antral LVA was quantitatively assessed offline as previously described and is depicted in Supporting Information Figure S1 [12]. First, the pulmonary vein (PV) ostia were identified based on points of maximal inflection between the PV wall and the left atrial (LA) wall. Next, the low‐voltage areas (LVA) around the ipsilateral PV ostia were delineated. These areas were projected in a posteroanterior (PA) view with a 30° superior tilt to ensure optimal visualization. The left and right isolated antral surface areas (IASA‐L and IASA‐R, cm²) were calculated by manually selecting and measuring the areas of the LVAs around the left and right PV ostia. The total antral scar area (IASA‐T) was determined as the sum of these measurements (IASA‐T = IASA‐L + IASA‐R).

### 2.4 Biomarkers of Myocardial Injury

Blood samples were collected in a fasting state on the morning before the procedure and 24 h postprocedure. High‐sensitivity troponin T (hs‐cTnT) was measured using the Roche Elecsys 2010 high‐sensitivity troponin T assay (Roche Diagnostics) with a 99th percentile cutoff of 14 ng/L and a corresponding coefficient of variation of 10% at 13 ng/L [10].

### 2.5 Postablation Management

A figure‐of‐eight suture was used to establish hemostasis, followed by the application of a pressure bandage and a subsequent 4‐h bed rest period. Following the ablation, all patients underwent transthoracic echocardiography to exclude the presence of a pericardial effusion. Oral anticoagulation was continued for a minimum of 2 months post‐intervention and was subsequently maintained based on the individual CHA₂DS₂‐VASc score. Antiarrhythmic medication prescribed before the catheter ablation was discontinued after the procedure.

### 2.6 Statistical Analysis

Continuous variables are described using median and interquartile range (IQR) and compared by Wilcoxon rank sum test. Categorical variables are presented as numbers as well as percentages and compared using χ2 or Fisher's exact test, as appropriate. Patients treated with the loop‐shaped catheter were compared to consecutive patients in the control group in a 1:3 ratio, all of whom also underwent mapping. A *p*‐value < 0.05 was considered statistically significant. Analysis was performed using R (R Core Team (2021), R Foundation for Statistical Computing, Vienna, Austria) and RStudio 2024.09.1 (RStudio Team (2019), RStudio Inc., Boston, MA, USA).

## 3 Results

### 3.1 Baseline Characteristics

A total of 120 patients were included, with 90 patients (75%) in the PCS group and 30 patients (25%) in the LCS group. The median age was 67 [59–73] years, with a significant difference between the groups (64 [59–70] in PCS vs. 72 [64–77] in LCS; *p* = 0.020). 29% of the patients were female. Paroxysmal AF was significantly less frequent in the PCS group (39%) compared to the LCS group (93%; *p* < 0.001), while persistent AF was more common in the PCS group (61% vs. 7%). The median LA diameter was slightly larger in the PCS group (42 [37–46] mm) compared with the LCS group (40 [33–42] mm; *p* = 0.044). Further baseline characteristics were comparable between the two groups and are summarized in Table 1.

**Table 1:** Baseline characteristics of patients undergoing PVI with either Farapulse or PulseSelect.

	Overall *N* = 120	PCS *N* = 90	LCS *N* = 30	*p* value
Age, years	67 [59−73]	64 [59−70]	72 [64−77]	0.020
Sex (female)	35 (29%)	26 (29%)	9 (30%)	0.908
BMI, kg/m^2^	27 [24−29]	27 [25−30]	25 [24−28]	0.037
Type of AF				< 0.001
Paroxysmal	63 (52%)	35 (39%)	28 (93%)	
Persistent	57 (48%)	55 (61%)	2 (7%)	
CHA_2_DS_2_‐VASc Score	2 [1−3]	2 [1−3]	2 [1−4]	0.441
LVEF, %	59 [53−63]	56 [50−62]	61 [59−63]	0.005
LA diameter, mm	41 [37−45]	42 [37−46]	40 [33−42]	0.044
EHRA score				0.175
I	38 (32%)	30 (33%)	8 (27%)	
IIa	31 (26%)	23 (26%)	8 (27%)	
IIb	30 (25%)	18 (20%)	12 (40%)	
III	15 (13%)	13 (14%)	2 (7%)	
IV	6 (5%)	6 (7%)	0 (0%)	
Hypertension	79 (66%)	60 (67%)	19 (63%)	0.739
Diabetes	15 (13%)	12 (13%)	3 (10%)	0.759
Hypercholesterolemia	42 (35%)	28 (31%)	14 (47%)	0.122
Coronary artery disease	10 (8%)	7 (8%)	3 (10%)	0.709
Heart failure	19 (16%)	13 (14%)	6 (20%)	0.564
History of Smoking	59 (49%)	48 (53%)	11 (37%)	0.114
Myocardial infarction	5 (4%)	3 (3%)	2 (7%)	0.598

Abbreviations: AF, atrial fibrillation; LA, left atrial; LCS, loop catheter system; LVEF, left ventricular ejection fraction; PVI, pulmonary vein isolation; PCS, pentaspline catheter system.

### 3.2 Procedural Characteristics

Overall, acute PVI was achieved in all patients. The median number of applications was 34 [32−38] and 34 [34−36] in the PCS and LCS groups, respectively. First pass isolation rate was 79% in the PCS group and 80% in the LCS group (*p* = 0.897). Procedural times were significantly shorter in the PCS group compared to the LCS group, including total procedure duration (57 [48−67] min vs. 66 [52−83] min, *p* = 0.016), LA dwell time (38 [32−48] min vs 54 [38−64] min, (*p* < 0.001), and ablation duration (17 [12−23] min vs. 24 [20−33] min, *p* < 0.001), respectively. Fluoroscopy time and dose were lower in the PCS group compared to the LCS group, with a fluoroscopy time of 11 [8−15] min and 14 [11–17] min (*p* = 0.020) and a dose of 534 [276−929] Gy·cm² versus 690 [506−1330] Gy·cm² (*p* < 0.001). Procedural characteristics are summarized in Table 2.

**Table 2:** Procedural characteristics of patients undergoing PVI with either PCS or LCS.

Variable	Overall *N* = 120	PCS *N* = 90	LCS *N* = 30	*p* value
Procedure duration, min	57 [49−68]	57 [48−67]	66 [52−83]	**0.016**
LA dwell time, min	40 [34−52]	38 [32−48]	54 [38−64]	**< 0.001**
Ablation duration, min	19 [14−24]	17 [12−23]	24 [20−33]	**< 0.001**
Fluoroscopy time, min	11 [8−15]	11 [8−15]	14 [11−17]	**0.020**
Fluoroscopy dose, Gycm^2^	586 [306−973]	534 [276−929]	690 [506−1330]	0.071
Number of applications	34 [32−37]	34 [32−38]	34 [34−36]	0.246
hs‐cTnT pre‐a blation, ng/l	10 [7−13]	10 [7−13]	10 [7−14]	0.668
hs‐cTnT postablation, ng/l	1390 [954−1936]	1282 [892−1894]	1588 [1281−2110]	**0.029**
Rhythm before ablation				**< 0.001**
AF	54 (45%)	50 (56%)	4 (13%)	
SR	66 (55%)	40 (44%)	26 (87%)	
FPI	95 (79%)	71 (79%)	24 (80%)	0.897
Complications	3 (3%)	3 (3%)	0 (0%)	0.572
Type of complications				> 0.999
Transient ischemic attack	1 (1%)	1 (1%)	0 (0%)	
Tamponade	1 (1%)	1 (1%)	0 (0%)	
Transient ST‐elevations	1 (1%)	1 (1%)	0 (0%)	

Abbreviations: AF, atrial fibrillation; FPI, first pass isolation; hs‐cTnT, high‐sensitivity cardiac troponin T; LCS, loop catheter system; PCS, pentaspline catheter system; PVI, pulmonary vein isolation; SR, sinus rhythm.

To address potential confounding from baseline differences, two additional analyses were performed. First, a sensitivity analysis in patients with paroxysmal AF (*n* = 63) showed results consistent with the overall cohort, including significantly shorter ablation duration in the PCS group (*p* < 0.001), while other procedural parameters remained comparable between groups (Supporting Information Table S2). Stratification by LAVI interquartile showed that procedural differences between PCS and LCS were preserved across all strata (Supporting Information Table S3).

### 3.3 Extent of Acute Antral Low Voltage Area

**Figure 1:**
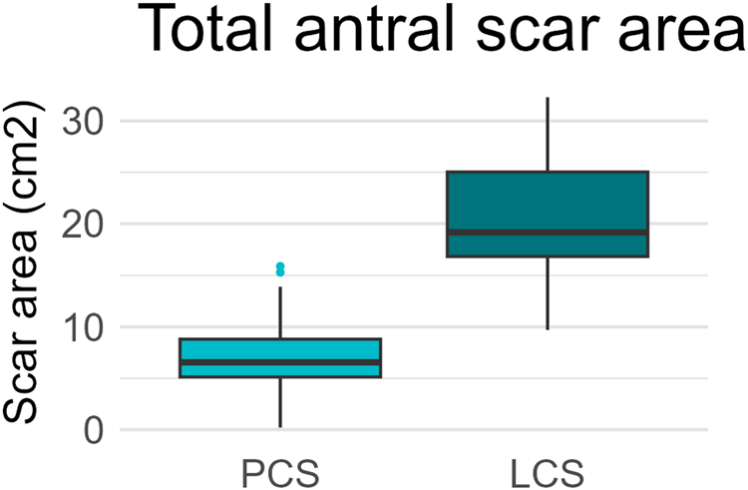
Boxplot comparing total scar area in a subgroup of patients undergoing PVI with either PCS or LCS. LCS, loop catheter system; PCS, pentaspline catheter system; PVI, pulmonary vein isolation.

The extent of acute antral LVA was assessed directly post PVI using a 3D‐EAM in 30 patients in each group. The extent of acute LVA following PVI was significantly different between the groups (Figure 1). Acute antral LVA was smaller in the PCS group compared with the LCS group (6.6 [5.0–8.9] cm² vs. 19.2 [16.8–25.4] cm², *p* < 0.001). This difference was consistent when analyzed separately for the left and right PV, with 3.7 [1.8–5.0] cm² versus 11.7 [8.3–12.7] cm², (*p* < 0.001) and 3.7 [1.4–4.9] cm² vs 8.3 [6.7–10.9] cm², *p* < 0.001 in the PCS versus LCS group respectively In the PCS group, 8 of 90 patients (8.9%) underwent PFA using a 35 mm catheter, while the remaining 82 patients (91.1%) were treated with a 31 mm catheter (Table 3).

**Table 3:** Scar area in a subgroup of patients undergoing PVI with either PCS or LCS, in combination with 3D‐EAM; PVI, PCS, and LCS.

	PCS *N* = 30	LCS *N* = 30	*p* value
Right‐sided scar area	3.7 [1.8−5.0]	11.7 [8.3−12.7]	**< 0.001**
Left‐sided scar area	3.7 [1.4−4.9]	8.3 [6.7−10.9]	**< 0.001**
Total scar area	6.6 [5.0−8.9]	19.2 [16.8−25.4]	**< 0.001**

Abbreviations: LCS, loop catheter system; PCS, pentaspline catheter system; PVI, pulmonary vein isolation.

### 3.4 Myocardial Injury

The pre‐ablation hs‐cTnT values were similar between groups, with a median of 10 [7−13] ng/L in the PCS group and 10 [7–14] ng/L in the LCS group (*p* = 0.668). Postablation troponin levels were elevated across both groups but significantly lower in the PCS group (1282 [892−1894] ng/L vs. 1588 [1281−2110] ng/L, *p* = 0.029). Correlation analysis between total LVA and hs‐cTnT levels showed no significant correlation in either group (PCS: r = 0.15, *p* = 0.434; LCS: r = 0.05, *p* = 0.796; Supporting Information Figure S2).

### 3.5 Procedural Safety

In the PCS group, one case of cardiac tamponade, one transient ischemic attack, and one transient ST elevation were observed, whereas no complications were reported in the LCS group. The cardiac tamponade was successfully treated with percutaneous drainage. The ST elevation occurred immediately after the transseptal puncture but before ablation and resolved spontaneously. One patient experienced diplopia following the procedure. Post‐procedural CT imaging ruled out bleeding or occlusion of the supraaortic vessels, and symptoms fully resolved within hours. Consequently, the event was classified as a transient ischemic attack (TIA).

### 3.6 Learning Curve

When comparing the first 10 and last 10 procedures in the PCS group, there were statistically significant changes in the respective procedural parameters. The LA dwell time (54 [44−55] min vs. 39 [32−44] min, *p* = 0.023) and the fluoroscopy dose 454 [367−667] Gycm^2^ versus 935 [670−2760] Gycm^2^, *p* = 0.015. When comparing the first 10 and last 10 procedures in the LCS group, in the circular PFA‐group there were no statistically significant changes in the respective procedural parameters.

## 4 Discussion

This study compared two PFA‐systems regarding procedural characteristics, acute antral LVA, and myocardial injury, in patients with AF undergoing de novo PVI. The key outcomes were as follow: First, the PCS system demonstrated shorter procedural times, including total procedure duration, left atrial dwell time, ablation duration, and fluoroscopic time. Nevertheless, the LCS exhibited procedural efficiency comparable to other state‐of‐the‐art single shot PFA devices, making it a viable alternative for de novo PVI. Second, the extent of acute antral LVA was significantly larger and more antral in the LCS group compared to the PCS group. Similarly, post‐procedural hs‐cTnT levels were significantly higher in the LCS group, reflecting a larger ablation area and potentially greater myocardial injury.

While multiple reports exist for each PFA system individually, comparative data between different PFA systems remains limited. Zylla et al. recently conducted a pilot study comparing acute procedural outcomes in 80 patients, with 40 treated with PCS and 40 with LCS. In contrast to this study, no significant differences in complication rates, procedural duration, or other procedural parameters were found. However, their study did not include 3D mapping for lesion assessment, nor did it evaluate hs‐TnT levels as a marker of myocardial injury, limiting direct comparison with our findings [13].

**Figure 2:**
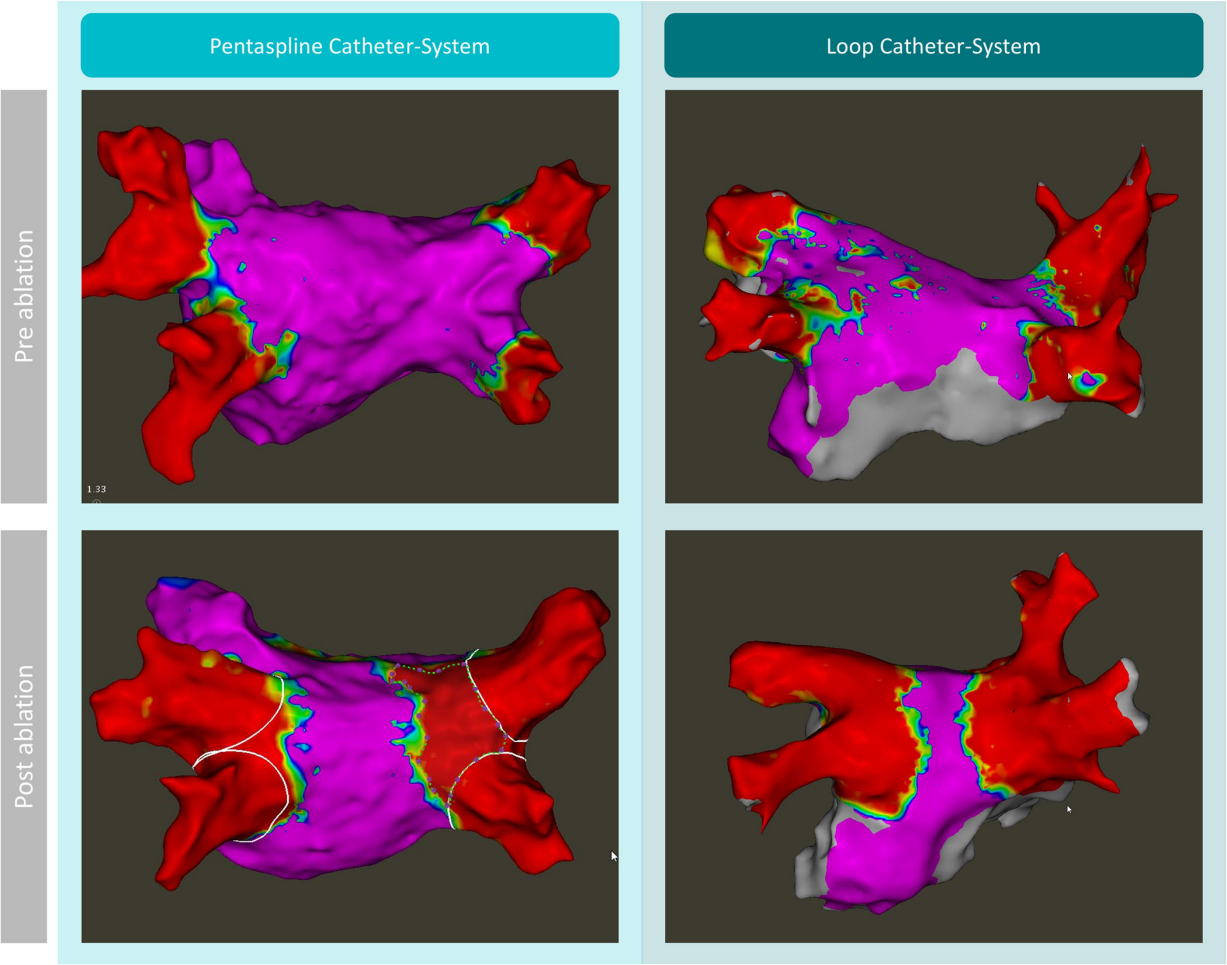
Preprocedural and post‐procedural electroanatomical mapping images before and after PVI. The left panel shows a bipolar voltage map of the left atrium in a posterior‐anterior view of a patient treated with a PCS. The right panel shows a bipolar voltage map of the left atrium in a posterior‐anterior view of a patient treated with the LCS. The white circles around the pulmonary veins mark the ostia of each vein. LCS, loop catheter system; PCS, pentaspline catheter system; PVI, pulmonary vein isolation.

The patient characteristics in this study were comparable to those reported in pivotal trials [4, 14]. The observation that LCS ablation results in extensive antral LVA is not unexpected given the suggested procedural workflow of the LCS (Figure 2). This workflow is supported by previously published data from the Pulsed AF pivotal trial [15] and consists of an ostial lesion set that targets the pulmonary vein ostium and an antral lesion set that targets the more distally antral region using the circular catheter. The optimal lesion set required for improved rhythm outcomes remains uncertain and will require future studies with long‐term follow‐up data. Similarly, for the PCS, our previous research suggests that reducing the number of applications might be sufficient to achieve similar clinical outcomes [16]. In the present study, both PFA systems were applied according to their respective standardized, manufacturer‐recommended workflows. New technologies such as the PCS and LCS often undergo modifications in their early phases of clinical use to maximize efficiency, efficacy, and safety. The hs‐cTnT levels following both ablation modalities demonstrated significantly higher levels in the LCS group. However, correlation analysis did not reveal a significant association between total LVA and hs‐cTnT levels (PCS: R = 0.15, *p* = 0.43; LCS: R = 0.049, *p* = 0.80; Supporting Information Figure S2). These findings suggest that troponin elevation may not directly reflect the extent of ablation lesions as assessed by voltage mapping, and the relationship between biomarker release, lesion characteristics, and long‐term durability remains uncertain [17].

Future studies incorporating advanced imaging and histopathological analysis are needed to further elucidate the relationship between lesion geometry, ablation system design, and biomarkers of myocardial injury. These investigations will help determine whether observed differences in troponin levels reflect comparable depths of myocardial injury or distinct lesion characteristics between the two PFA systems.

Although the PCS system requires a larger catheter and sheath (12 F and 13 F vs. 9 F and 10 F for LCS), no vascular complications occurred in our cohort. This aligns with large‐scale data from trials like PULSED AF (0% major vascular events in 300 patients) and the MANIFEST‐17K registry (0.3% in over 17 000 patients using a 13 F system) [14, 18]. In our study, all access was ultrasound‐guided and performed by experienced operators, further supporting procedural safety.

Some limitations should be acknowledged. First, this was a non‐randomized, observational single‐center study with all its limitations. Second, the statistical power was limited due to the relatively small sample size. Third, we did not include a variable‐loop circular catheter, which has now received FDA approval [19]. Fourth, follow‐up data on arrhythmia recurrence, PV reconnection, and lesion durability were not available. Thus, the predictive value of LVA and hs‐cTnT as surrogate markers for long‐term efficacy remains to be established. Studies comparing a variable‐loop circular catheter with PCS or LCS are currently lacking and warrant further investigation.

## 5 Conclusion

Both the PCS and LCS represent highly efficient tools for PVI. While the PCS was associated with significantly shorter procedural times, the LCS resulted in more extensive antral LVA and higher levels of myocardial injury. Further studies with long‐term follow‐up are needed to evaluate the clinical implications of procedural differences, particularly regarding durability of LVA and long‐term rhythm outcomes.

## Supporting information


**Figure S1:** Post‐ablation left atrial voltage anteral scar area measurement: Superior (Left) and posterior (Right) views of the voltage map, color‐coded with magenta (≥ 0.5 mV) and red (≤ 0.1 mV).

**Figure S2:** Correlation between total antral low‐voltage area and hs‐cTnT levels in PCS and LCS groups.

Supporting Appendix PulseSelect Farapulse 1.

Supporting Appendix PulseSelect Farapulse 2.

## Data Availability

The data that support the findings of this study are available from the corresponding author upon reasonable request.
