# Supplementary figures and images for: Comparison of Two Different Pulsed Field Ablation Systems: The Dual Pulse System Study
Source: J Cardiovasc Electrophysiol. 2025 Sep 19;36(11):2955–62. doi: 10.1111/jce.70078 (PMC12614143; doi:10.1111/jce.70078)

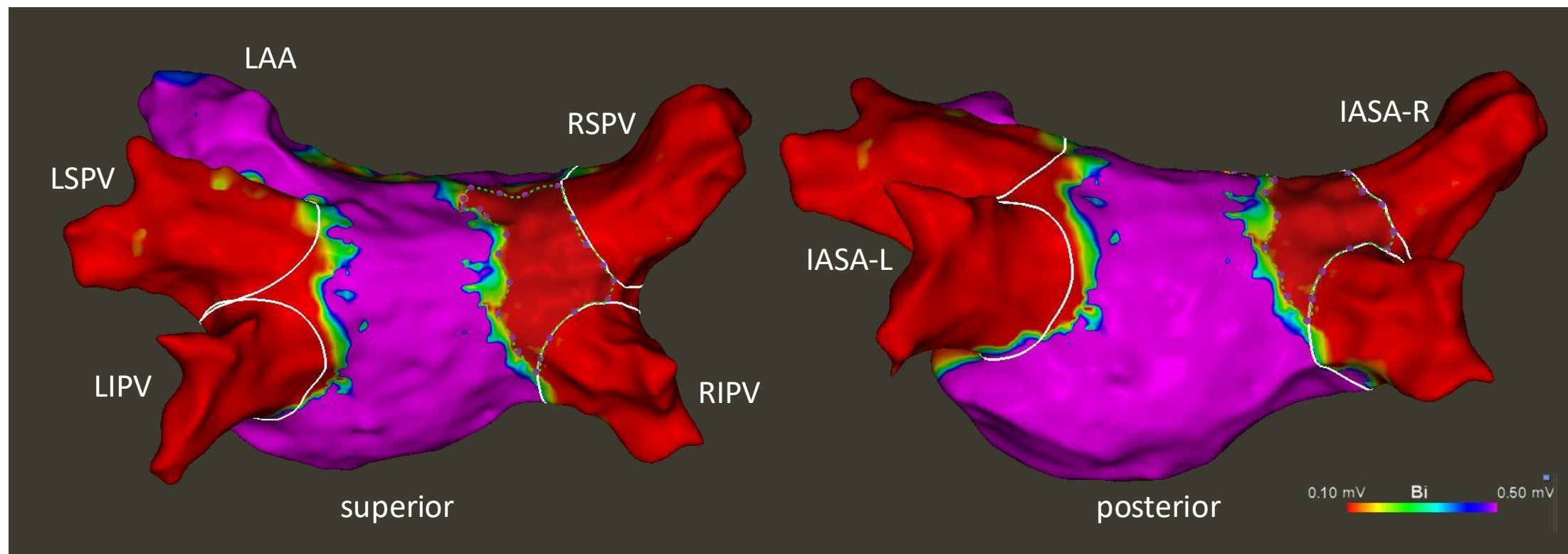

Supplement: Supplementary file 1 — Figure S1: Post‐ablation left atrial voltage anteral scar area measurement: Superior (Left) and posterior (Right) views of the voltage map, color‐coded with magenta (≥ 0.5 mV) and red (≤ 0.1 mV). [file JCE-36-2955-s004.pdf]

# Pearson correlation: Total scar area vs. hs-cTnT

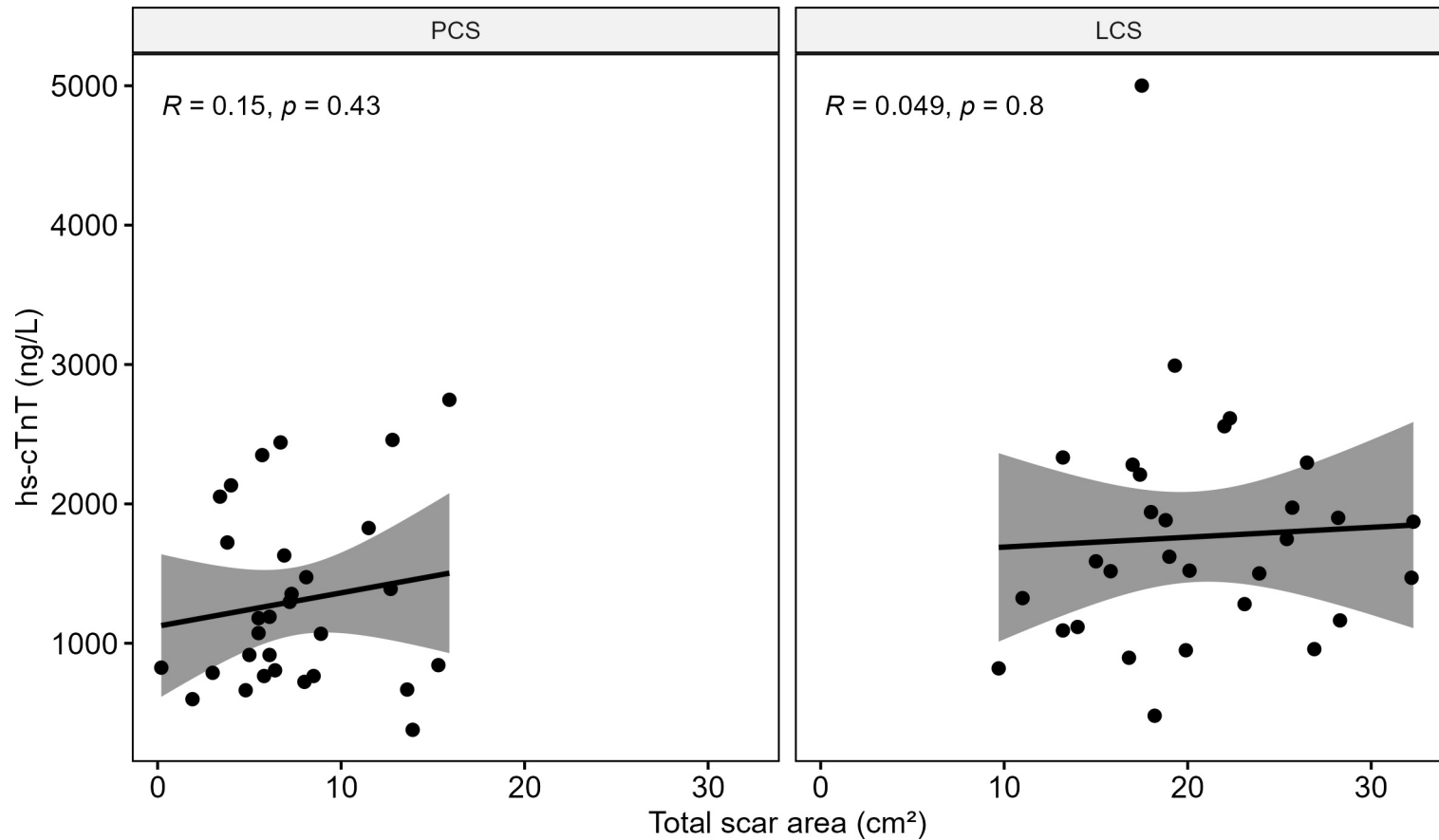

Supplement: Supplementary file 2 — Figure S2: Correlation between total antral low‐voltage area and hs‐cTnT levels in PCS and LCS groups. [file JCE-36-2955-s001.pdf]
