# Supplementary material for: Comparison of Two Different Pulsed Field Ablation Systems: The Dual Pulse System Study
Source: J Cardiovasc Electrophysiol. 2025 Sep 19;36(11):2955–62. doi: 10.1111/jce.70078 (PMC12614143; doi:10.1111/jce.70078)
Supplement: Supplementary file 3 — Supporting Appendix PulseSelect Farapulse 1. [file JCE-36-2955-s003.docx]

Supplementary Appendix

Table of content

[Figure S1 2](#_Toc200548081)

[Figure S2 3](#_Toc200548082)

[Table S1 4](#_Toc200548083)

[Table S2 5](#_Toc200548084)

[Table S3 6](#_Toc200548085)

# Figure S1
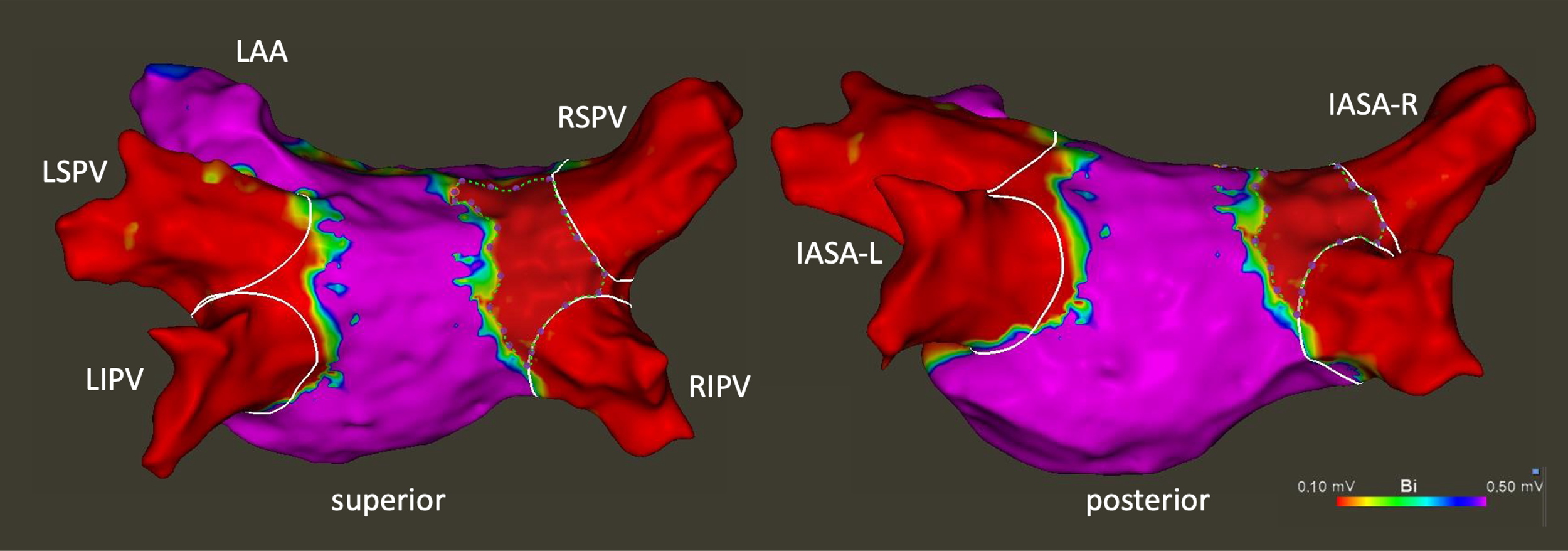


**Figure S1:** Post-ablation left atrial voltage anteral scar area measurement: Superior (Left) and posterior (Right) views of the voltage map, color-coded with magenta (≥0.5 mV) and red (≤0.1 mV). First, the ostium of each PV was defined (white circles around the RSPV, RIPV, LSPV, LIPV). Subsequently, the left and right isolated antral scar areas (IASA-L and IASA-R) were manually measured as low-voltage areas (red) and and the total isolated antral scar area (IASA-T) was calculated. The non-ablated area is the magenta area between the low voltage area. LAA = Left atrial appendage; LIPV = Left inferior pulmonary vein; LSPV = Left superior pulmonary vein, PV = Pulmonary vein; RIPV = Right inferior pulmonary vein; RSPV = Right superior pulmonary vein.

# Figure S2


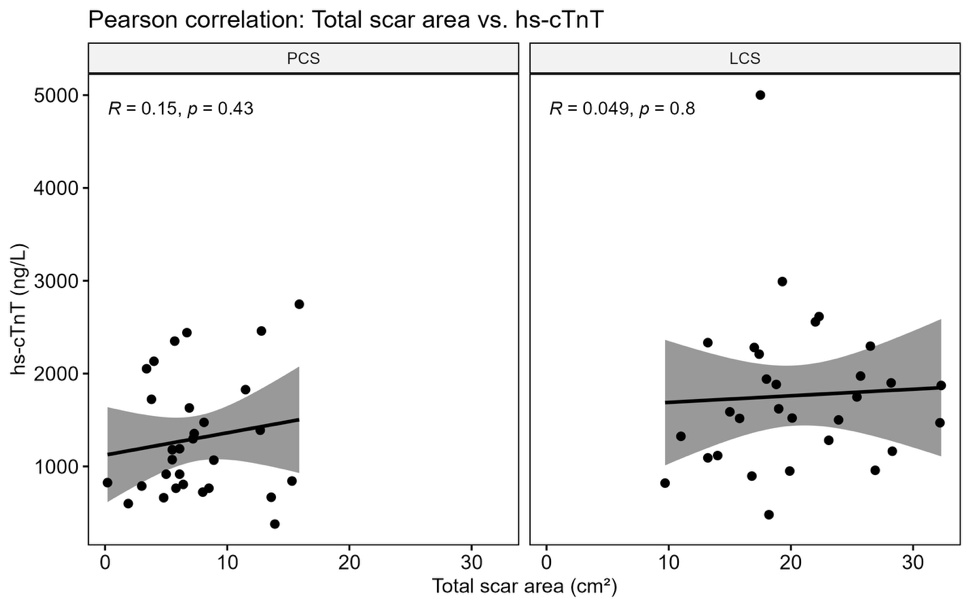


**Figure S2:** Correlation between total antral low-voltage area and hs-cTnT levels in PCS and

LCS groups. PCS = Pentaspline catheter system; LCS = loop catheter system.

# Table S1

|  | **Pentaspline catheter-system** | **Loop catheter-system** |
| --- | --- | --- |
| **Brand name** | FARAPULSE  (Boston Scientific) | PulseSelect  (Medtronic) |
| **Ablative energy** | Short, high voltage pulses | Short, high voltage pulses |
| **Energy delivery** | 20 electrodes | 9 electrodes |
| **Deployed size (diameter)** | 31/ 35 mm | 25 mm |
| **Electrodes for electrogram recording and pacing** | 5 electrodes of 2 mm size | 9 electrodes of 3 mm size |
| **Inter-electrode spacing** | 17 mm (standard) | 3,75 mm |
| **Typical duration of a single application** | 2.5 sec with 5 pulses | 4 packets of pulses, each lasting 100 to 200 ms |
| **Typical count application per PV** | 4x “Flower” configuration  4x “Basked” configuration | 4 x antral applications  4 x ostial applications |
| **Delivery sheath** | 13-F | 10-F |
| **CE mark** | 02/2021 | 11/2023 |
| **FDA Approval** | 01/2024 | 12/2023 |

**Table S1**: Technical characteristics of Farapulse and PulseSelect PFA catheter. FDA = Food and Drug Administration; PFA = Pulsed-field ablation.

# Table S2

| **Variable** | **Overall** N = 63*^1^* | **PCS** N = 35*^1^* | **LCS** N = 28*^1^* | **p-value***^2^* |
| --- | --- | --- | --- | --- |
| **Total Procedure Duration, min** | 59 [50 - 74] | 58 [50 - 68] | 66 [50 - 85] | 0.280 |
| **LA dwell time, min** | 42 [36 - 56] | 40 [36 - 53] | 51 [37 - 65] | 0.067 |
| **Ablation Duration, min** | 23 [17 - 30] | 20 [15 - 27] | 24 [21 - 33] | **0.010** |
| **Fluoroscopy time, min** | 12 [10 - 17] | 12 [10 - 16] | 14 [10 - 17] | 0.478 |
| **Fluoroscopy dose, Gycm^2^** | 585 [304 - 990] | 447 [251 - 1,041] | 608 [505 - 988] | 0.152 |
| **Number of Applications** | 34 [32 - 36] | 33 [32 - 39] | 35 [34 - 36] | 0.185 |
| **Troponin Pre Ablation** | 10 [7 - 14] | 10 [7 - 16] | 9 [7 - 13] | 0.496 |
| **Troponin Post Ablation** | 1,726 [1,199 - 2,192] | 1,759 [1,068 - 2,192] | 1,621 [1,281 - 2,281] | 0.801 |
| **Rhythm before Intervention** |  |  |  | 0.277 |
| AF | 9 (14%) | 7 (20%) | 2 (7%) |  |
| SR | 54 (86%) | 28 (80%) | 26 (93%) |  |
| **FPI** | 54 (86%) | 31 (89%) | 23 (82%) | 0.494 |
| **Complications** | 2 (3%) | 2 (6%) | 0 (0%) | 0.498 |
| **Type of complications** |  |  |  | >0.999 |
| none | 61 (97%) | 33 (94%) | 28 (100%) |  |
| Tamponade | 1 (2%) | 1 (3%) | 0 (0%) |  |
| Transient ST-elevation | 1 (2%) | 1 (3%) | 0 (0%) |  |
| *^1^*Median [Q1 - Q3]; n (%) | | | | |
| *^2^*Wilcoxon rank sum test; Wilcoxon rank sum exact test; Fisher's exact test | | | | |

**Table S2:** Sensitivity analysis of procedural characteristics in patients with paroxysmal atrial fibrillation (PAF), comparing pulsed field ablation using the pentaspline catheter system (PCS) and the loop catheter system (LCS). LA = Left atrial; AF = Atrial Fibrillation; SR = Sinus rhythm; FPI = First pass isolation

# Table S3

|  | **small/mild enlargement** | | | **moderately enlarged** | | | **severely enlarged** | | |
| --- | --- | --- | --- | --- | --- | --- | --- | --- | --- |
| **Variable** | **PCS** N = 44*^1^* | **LCS** N = 18*^1^* | **p-value***^2^* | **PCS** N = 34*^1^* | **LCS** N = 11*^1^* | **p-value***^2^* | **PCS** N = 12*^1^* | **LCS** N = 1*^1^* | **p-value***^3^* |
| **Total Procedure Duration, min** | 55 [47 - 67] | 58 [49 - 78] | 0.200 | 58 [48 - 65] | 73 [66 - 91] | **0.021** | 56 [51 - 63] | 66 [66 - 66] | 0.462 |
| **LA dwell time, min** | 37 [33 - 50] | 43 [37 - 61] | **0.031** | 40 [32 - 48] | 56 [49 - 73] | **0.009** | 40 [34 - 43] | 54 [54 - 54] | 0.181 |
| **Ablation Duration, min** | 18 [12 - 23] | 23 [20 - 32] | **0.003** | 16 [11 - 21] | 27 [24 - 33] | **0.001** | 19 [15 - 25] | 18 [18 - 18] | >0.999 |
| **Fluoroscopy time, min** | 11 [9 - 15] | 12 [10 - 15] | 0.877 | 9 [8 - 13] | 15 [14 - 20] | **0.002** | 12 [7 - 15] | 14 [14 - 14] | 0.562 |
| **Fluoroscopy dose, Gycm^2^** | 408 [256 - 821] | 577 [467 - 896] | 0.229 | 585 [346 - 1,047] | 992 [758 - 2,165] | **0.049** | 708 [517 - 1,454] | 2,090 [2,090 - 2,090] | 0.333 |
| **Number of Applications** | 34 [32 - 36] | 36 [34 - 36] | **0.024** | 35 [34 - 38] | 34 [34 - 36] | 0.586 | 33 [32 - 38] | 24 [24 - 24] | 0.119 |
| **Troponin Pre Ablation** | 9 [6 - 13] | 10 [7 - 14] | 0.451 | 11 [8 - 13] | 7 [7 - 16] | 0.516 |  |  | >0.999 |
| **Troponin Post Ablation** | 1,641 [1,002 - 2,052] | 1,605 [1,324 - 2,110] | 0.447 | 1,068 [892 - 1,354] | 1,697 [896 - 2,296] | 0.093 | 1,216 [697 - 1,804] | 950 [950 - 950] | 0.769 |
| **Rhythm before Intervention** |  |  | **0.015** |  |  | **0.007** |  |  | 0.308 |
| AF | 19 (43%) | 2 (11%) |  | 22 (65%) | 2 (18%) |  | 9 (75%) | 0 (0%) |  |
| SR | 25 (57%) | 16 (89%) |  | 12 (35%) | 9 (82%) |  | 3 (25%) | 1 (100%) |  |
| **FPI** | 39 (89%) | 14 (78%) | 0.427 | 22 (65%) | 9 (82%) | 0.458 | 10 (83%) | 1 (100%) | >0.999 |
| *^1^*Median [Q1 - Q3]; n (%) | | | | | | | | | |
| *^2^*Wilcoxon rank sum test; Wilcoxon rank sum exact test; Pearson's Chi-squared test; Fisher's exact test | | | | | | | | | |
| *^3^*Wilcoxon rank sum exact test; Wilcoxon rank sum test; Fisher's exact test | | | | | | | | | |

**Table S3:** Comparison of procedural parameters between PCS and LCS systems across LAVI-based interquartile strata (mild, moderate, and severe enlargement), assessing consistency of findings in relation to atrial size. LA = Left atrial; AF = Atrial Fibrillation; SR = Sinus rhythm; FPI = First pass isolation.
