# Supplementary material for: Comparison of Two Different Pulsed Field Ablation Systems: The Dual Pulse System Study
Source: J Cardiovasc Electrophysiol. 2025 Sep 19;36(11):2955–62. doi: 10.1111/jce.70078 (PMC12614143; doi:10.1111/jce.70078)
Supplement: Supplementary file 4 — Supporting Appendix PulseSelect Farapulse 2. [file JCE-36-2955-s002.docx]

Supplementary Appendix

Table of contents

[Figure S1 2](#_Toc189655845)

[Table S1 3](#_Toc189655846)

# Figure S1
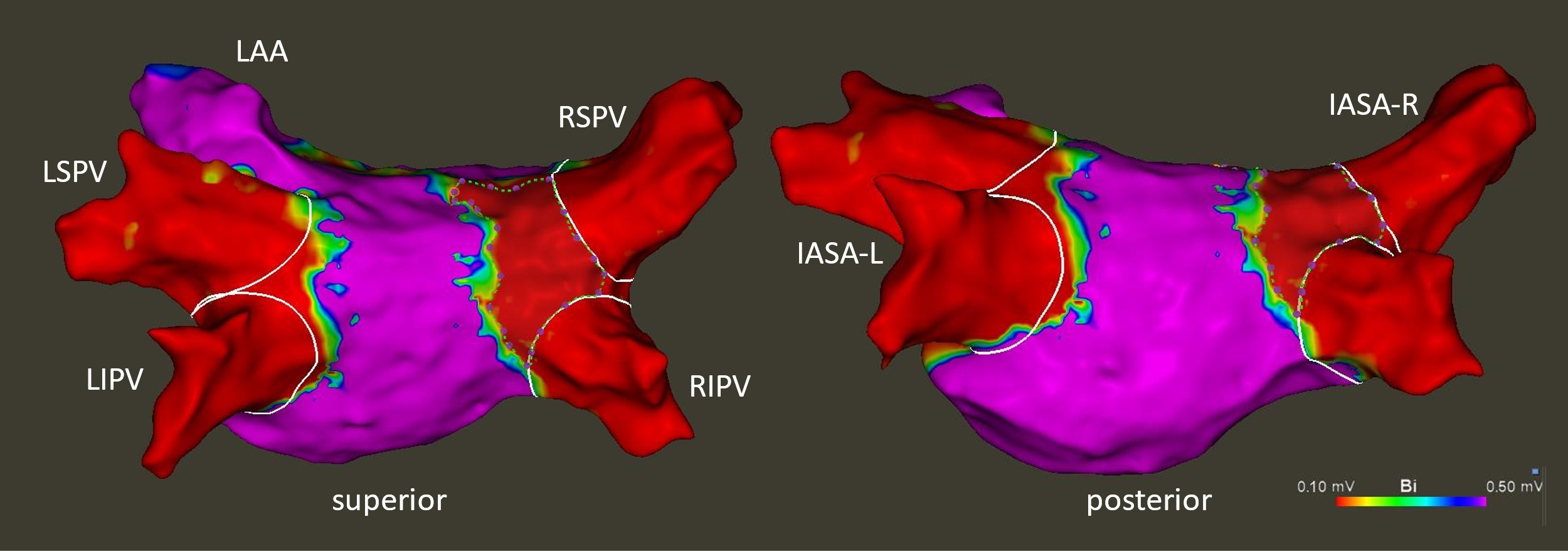


**Figure S1:** Post-ablation left atrial voltage anteral scar area measurement: Superior (Left) and posterior (Right) views of the voltage map, color-coded with magenta (≥0.5 mV) and red (≤0.1 mV). First, the ostium of each PV was defined (white circles around the RSPV, RIPV, LSPV, LIPV). Subsequently, the left and right isolated antral scar areas (IASA-L and IASA-R) were manually measured as low-voltage areas (red) and and the total isolated antral scar area (IASA-T) was calculated. The non-ablated area is the magenta area between the low voltage area. LAA = Left atrial appendage; LIPV = Left inferior pulmonary vein; LSPV = Left superior pulmonary vein, PV = Pulmonary vein; RIPV = Right inferior pulmonary vein; RSPV = Right superior pulmonary vein.

# Table S1

|  | **Pentaspline catheter-system** | **Loop catheter-system** |
| --- | --- | --- |
| **Brand name** | FARAPULSE  (Boston Scientific) | PulseSelect  (Medtronic) |
| **Ablative energy** | Short, high voltage pulses | Short, high voltage pulses |
| **Energy delivery** | 20 electrodes | 9 electrodes |
| **Deployed size (diameter)** | 31/ 35 mm | 25 mm |
| **Electrodes for electrogram recording and pacing** | 5 electrodes of 2 mm size | 9 electrodes of 3 mm size |
| **Inter-electrode spacing** | 17 mm (standard) | 3,75 mm |
| **Typical duration of a single application** | 2.5 sec with 5 pulses | 4 packets of pulses, each lasting 100 to 200 ms |
| **Typical count application per PV** | 4x “Flower” configuration  4x “Basked” configuration | 4 x antral applications  4 x ostial applications |
| **Delivery sheath** | 13-F | 14-F |
| **CE mark** | 02/2021 | 11/2023 |
| **FDA Approval** | 01/2024 | 12/2023 |

**Table S1**: Technical characteristics of Farapulse and PulseSelect PFA catheter. FDA = Food and Drug Administration; PFA = Pulsed-field ablation.
